# Spanish Validation of the Leader Empowering Behavior Questionnaire (LEBQ)

**DOI:** 10.3389/fpsyg.2019.02368

**Published:** 2019-10-18

**Authors:** Tomas Bonavia, Juan A. Marin-Garcia

**Affiliations:** ^1^Departamento de Psicología Social, Universitat de València, Valencia, Spain; ^2^ROGLE – Departamento de Organización de Empresas, Universitat Politècnica de València, Valencia, Spain

**Keywords:** empowering leader behavior, leadership empowerment behavior, scale validation, instrumental study, human resource management, continuous improvement, job satisfaction

## Abstract

The concept of empowering leadership (EL) has attracted widespread academic and practical interest and different questionnaires have been developed to measure it. However, there are no instruments to measure EL in the Spanish language. This article presents the translation, adaptation, and validation of a scale to measure this construct. In addition, it analyzes the relationship between managers’ EL and employees’ job satisfaction. In turn, the study analyzes whether employees who participate in a greater number of continuous improvement (CI) programs have supervisors who favor more empowering behaviors. A total of 739 participants with various occupations from different companies that have implemented CI processes filled out the Spanish version of the Leader Empowering Behavior Questionnaire (LEBQ-sp). Two different subsamples were used to test the relationships between the LEBQ and job satisfaction and CI, by means of Pearson’s correlation coefficient and analysis of variance, making it possible to provide evidence about the validity of the Spanish LEBQ. The confirmatory factor analysis supported the original structure of the six-factor model. The factors show a high level of internal consistency, as well as sufficient convergent and discriminant validity. Moreover, the results show that the more companies invest in formal CI programs, the more important it is for their leaders to adapt their behavior by displaying more EL. The LEBQ-sp is a valid and reliable instrument for use in research and a useful tool for applied purposes in the context of Spanish-speaking countries.

## Introduction

Since it was introduced in the 1980s, empowerment has become a popular management practice. In modern societies, it has been shown to be an effective tool to improve both public and private companies by increasing the quality of the services they offer, their productivity, or the necessary internal processes for their good functioning (March, 2011; Sharma and Kirkman, 2015; Lee et al., 2018).

Two major perspectives on the empowerment phenomenon have emerged (Seibert et al., 2011; Lee et al., 2018). The first perspective conceives empowerment as a set of structures, policies, and practices designed to decentralize power and authority throughout the organization (structural empowerment). The second perspective, which is more psychological, focuses on the effects of these practices on employees’ initiative and motivation (psychological empowerment). Among the former, one development that has gained importance over time is empowering leadership (EL), which can be defined as the set of “behaviors that share power with subordinates” (Vecchio et al., 2010, p. 531). Although EL has conceptual similarities with transformational leadership and leader–member exchange (Sharma and Kirkman, 2015), studies have shown that they are different constructs (Amundsen and Martinsen, 2014), and the results largely reveal that EL shows incremental predictive validity over the other two leadership models (Lee et al., 2018).

Most of the research on EL has almost exclusively focused on its positive outcomes (Sharma and Kirkman, 2015). Moreover, Arnold et al. (2000) noted that there is little research on the role of effective empowering leader behavior, possibly due to the limited interest shown by the research in identifying leaders’ empowering behaviors (Konczak et al., 2000). Consequently, only a few scales have been published that measure what EL really consists of Amundsen and Martinsen (2014). Among them, only three scales measure EL at the individual level (the others focus on teamwork at the group level in less hierarchical and more decentralized organizations): the Leader Empowering Behavior Questionnaire (LEBQ) developed by Konczak et al. (2000), the scale developed by Ahearne et al. (2005), and the Empowering Leadership Scale (ELS) developed by Amundsen and Martinsen (2014).

The previous observation, as Amundsen and Martinsen (2014, p. 488) point out: “highlights the importance of studying EL at the individual level in more traditional hierarchical structures wherein leaders relate to individual employees to a greater extent than teams.” The LEBQ addresses this need for instruments in cultural contexts where a hierarchical supervisor–employee relationship is still common, as in Spanish-speaking countries (Romero and Pérez, 2003; Tàpies, 2011). Therefore, the goals that guide the current study are: first, to carry out the Spanish adaptation of the LEBQ, one of the few existing scales to measure the empowerment behavior of bosses toward their subordinates; and, second, to show the relationships between these EL behaviors and job satisfaction and continuous improvement (CI).

As occurs with the LEBQ, none of the following scales to measure EL has been validated in Spanish: The Self-Management Leadership Questionnaire (SMLQ) developed by Manz and Sims (1987); the Strategic Leadership Questionnaire II (SLQII) originated by Cox and Sims (1996) and later analyzed by Pearce and Sims (2002); and the ELS developed by Amundsen and Martinsen (2014). Of the remaining instruments, there is a partial adaptation to Spanish (Martínez-Córcoles, 2012) and a complete first adaptation stemming from a master’s thesis (Eloíza Becerra et al., 2017) of the Empowering Leadership Questionnaire (ELQ) developed by Arnold et al. (2000); for the scale developed by Ahearne et al. (2005), an adaptation already exists (Huertas-Valdivia et al., 2018). All of this justifies the relevance of the present study.

The LEBQ was designed for a leadership development training program in order to evaluate behavioral indicators exhibited during individual-level interactions between a supervisor and subordinate. It has been used in a variety of studies, for example, to measure EL, followers’ feedback-seeking, and task performance (Qian et al., 2018), or the leadership behaviors of school superintendents and their relationship with student performance (Zigun, 2016). The scale has been translated into various languages, such as Turkish or Estonian, and applied in different cultural contexts, such as South Africa or Asia. However, we have not found an adaptation of this questionnaire in Spanish.

The LEBQ has shown acceptable internal consistency. Konczak et al. (2000) found that all the alpha reliability coefficients for the scores in the six-factor model were good (range = 0.82–0.90). The inter-factor correlations ranged from 0.40 to 0.88. Various researchers have found reliability coefficients >0.90 for the total scale and acceptable construct validity of the questionnaire (Stander, 2007). Konczak et al. (2000) found significant correlations between the dimensions of the LEBQ and job satisfaction (ranging from 0.32 with accountability to 0.63 for delegation of authority). These significant correlations were also found between leader empowering behavior and intrinsic (*r* = 0.64) and extrinsic (*r* = 0.81) job satisfaction (Stander and Rothmann, 2009).

On the other hand, Fryer et al. (2007) argued that employee empowerment is a critical factor in CI success, and he is not the only author to state this (Hirzel et al., 2017). CI can be defined as a planned, organized, and systematic process of incremental and ongoing change (Garcia-Sabater et al., 2012; Hirzel et al., 2017; Jurburg et al., 2017). To carry out these changes, the process should be extended throughout an organization, it should also form part of the day-to-day functions of the company and be of a voluntary nature, and it should be sustainable in time. The aim of CI is to achieve a reduction in costs or an enhancement of quality, flexibility, or productivity (Garcia-Sabater et al., 2012). Both academics and practitioners agree that CI is a difficult process to implement, that it requires profound changes, and that a high level of participation and commitment is necessary in order for these initiatives to be successful.

If we want workers to participate actively in the improvement system, we must lead them in a way that foments their trust and commitment (Jurburg et al., 2017). EL has been proposed as one of the types of leadership that can achieve this, obtaining empirical support (Van Assen, 2018) based on the use of the LEBQ (Costa Nogueira et al., 2018). The role of the manager changes in contexts where CI and other related techniques are implemented (Poksinska et al., 2013). The focus changes from managing processes to managing people, and the primary role of managers is now to motivate, coach, and develop individuals and teams. In summary, the key question is for managers to ultimately focus on empowering employees and involving them in CI activities (Hirzel et al., 2017). Therefore, a close relationship can be detected between CI and EL.

The LEBQ contains 17 items grouped in six dimensions (three items per construct, except for one of them). Delegation of authority refers to whether the leaders grant power to subordinates. Accountability for outcomes addresses the leader’s emphasis on taking responsibility for consequences. Self-directed decision making implies that the leader encourages independent decision-making. Information sharing evaluates whether the leaders share information and knowledge with the employees. Skill development is concerned with the extent to which the leader facilitates the development of skills and secures appropriate training for employees. Coaching for innovative performance is related to behavior that encourages calculated risk-taking and new ideas and provides performance feedback to employees, treating their mistakes and setbacks as opportunities to learn (Konczak et al., 2000). The LEBQ is answered on a Likert-type scale that ranges from 1 (“strongly disagree”) to 7 (“strongly agree”). Higher scores indicate higher employee perceptions of leader empowering behaviors.

Nearly all the scales to measure EL include various sub-dimensions (Amundsen and Martinsen, 2014). However, the vast majority of the empirical research treats these scales as unidimensional (Lee et al., 2018). Konczak et al. (2000) tested the fit of its model through confirmatory factor analyses (CFAs) in two sub-samples of 1,309 subordinates, all of whom belonged to a single organization. In both cases, they obtained support for a six-dimensional model of the LEBQ and demonstrated a poor fit of the single-factor model. Koçak and Burgaz (2017) also obtained support for the multifactorial structure of this scale through CFA in two different studies. However, Stander and Rothmann (2009) defended a one-dimensional structure of their modified version of the LEBQ, although through a simple principal components analysis. Other authors have defended a three-factor model (see Mendes and Stander, 2011).

## Materials and Methods

### Participants and Procedure

Purposive snowball sampling was used to collect employees with a broad set of occupations from different companies (Saunders et al., 2016), the majority of which were involved in the implementation of CI processes. In all, 739 workers were finally interviewed face-to-face by students from a CI course in an industrial engineering master’s degree program. Their participation was voluntary, the answers were anonymous, the written consent was obtained from the participants before the interview, and the participants did not receive any monetary compensation. These students previously received 4 weeks (10 h of training) of instruction about the contents of the interview and the way it would be carried out. Of the people interviewed, 651 provided complete data for all the items on the LEBQ-sp (53% of the participants were men and 47% women). The ages ranged from 16 to 65 years, with a mean age of 35 years (*SD* = 10.3) and a mean of 7.5 years working in their current company (*SD* = 8.3). In addition, 48% of the participants worked for service companies, 21% worked in industry, and the rest worked in construction, public companies, and other sectors (31%). Datasets are available on request.

To avoid common method bias (CMB), we do not include complex items, we use a different choice of scale anchors for the LEBQ and MSQ items, we eliminate reverse-coded items, and we avoid prime effects (Podsakoff et al., 2003; Schwarz et al., 2017). In addition, we verified *a posteriori* that there was no evidence of CMB by using Harman’s single-factor test or the measured latent marker variable (MLMV) approach (Chin et al., 2013; Schwarz et al., 2017).

### Instrument

The LEBQ was designed by Konczak et al. (2000) to measure the leader behaviors that promote empowerment among the subordinates (Table 1). To translate the scale into Spanish, we followed the usual protocol for this type of task (Doval Dieguez and Viladrich Segués, 2011; Muñiz et al., 2013; Llosa et al., 2017). The authors of the study carried out the initial translation of the items from English to Spanish. At this point, there was discussion about the idea underlying Factor 2, “Accountability,” that is, “making employees responsible” for the tasks assigned and the results obtained. The concern was that it might not be completely understood in our cultural context. In Spain, it is said that people are more likely to assume that the final responsibility for the goals reached would fall on the supervisors and not on the operators. This phenomenon is commonly observed in family companies, which, in our country, make up 85% of all companies (Tàpies, 2011). This situation even extends to Spanish cooperatives, where we find that the workers participate less in the management of their organizations than other collectives (Romero and Pérez, 2003). Transferring this responsibility to the employees would excuse the bosses from one of their most important obligations, namely, to answer to the degree to which the established objectives are met. Therefore, the decision was made to adapt these items to indicate the degree to which the supervisors keep their subordinates informed. Later, a native English-speaking translator and three bilingual experts translated the Spanish items back into English (without being familiar with the original instrument). The results confirmed the equivalence of the two versions, except for Factor 2, as explained above.

**TABLE 1:** LEBQ factors and items.

**Item**	**Original English version (Konczak et al., 2000)**	**FL**	**Spanish version (LEBQ-sp)**
	**F1 Delegation of authority**		**Delegación de autoridad**
LEBQ01	1. My manager gives me the authority I need to make decisions that improve work processes and procedures	0.86	01 Tu jefe/a te proporciona la autoridad que necesitas para mejorar los procesos o procedimientos de tu trabajo
LEBQ02	2. My manager gives me the authority to make changes necessary to improve things	0.86	02 Tu jefe/a te proporciona autoridad para realizar los cambios necesarios para mejorar las cosas
LEBQ03	3. My manager delegates authority to me that is equal to the level of responsibility that I am assigned	0.83	03 La autoridad que tu jefe/a delega en ti es proporcional al grado de responsabilidad que se te asigna en las tareas
	**F2 Accountability**		**Ser informado/a**
LEBQ04	4. My manager holds me accountable for the work I am assigned	0.87	04 Tu jefe/a te informa de las tareas que debes hacer
LEBQ05	5. I am held accountable for performance and results	0.91	05 Tu jefe/a te informa de tu rendimiento y los resultados de la unidad
LEBQ06	6. My manager holds people in the department accountable for customer satisfaction	0.67	06 Tu jefe/a informa a las personas de la unidad de los niveles de satisfacción de los clientes
	**F3 Self-directed decision making**		**Toma de decisiones autodirigida**
LEBQ07	7. My manager tries to help me arrive at my own solutions when problems arise, rather than telling me what he/she would do	0.81	07 Tu jefe/a intenta ayudarte para que generes tus propias soluciones a los problemas que surgen, en lugar de decirte que es lo que él/ella haría
LEBQ08	8. My manager relies on me to make my own decisions about issues that affect how work gets done	0.79	08 Tu jefe/a confía en ti para que tomes tus propias decisiones sobre asuntos que afectan a cómo se hace el trabajo
LEBQ09	9. My manager encourages me to develop my own solutions to problems I encounter in my work	0.87	09 Tu jefe/a te anima a que desarrolles tus propias soluciones a los problemas que encuentras en tu trabajo
	**F4 Information sharing**		**Intercambio de información**
LEBQ10	10. My manager shares information that I need to ensure high quality results	0.88	10 Tu jefe/a comparte contigo la información que necesitas para asegurar resultados de elevada calidad
LEBQ11	11. My manager provides me with the information I need to meet customers’ needs	0.96	11 Tu jefe/a te facilita la información que necesitas para satisfacer las necesidades de vuestros clientes
	**F5 Skill development**		**Desarrollo de habilidades**
LEBQ12	12. My manager encourages me to use systematic problem-solving methods (e.g., the seven-step problem-solving model)	0.55	12 Tu jefe/a te anima a que uses métodos formales de resolución de problemas (por ejemplo el modelo de siete pasos para la resolución de problemas)
LEBQ13	13. My manager provides me with frequent opportunities to develop new skills	0.88	13 Tu jefe/a te proporciona muchas oportunidades para desarrollar nuevas habilidades
LEBQ14	14. My manager ensures that continuous learning and skill development are priorities in our department	0.86	14 Tu jefe/a garantiza que el aprendizaje continuo y el desarrollo de habilidades sean una de las prioridades de vuestro departamento
	**F6 Coaching for innovative performance**		**Entrenamiento para la innovación**
LEBQ15	15. My manager is willing to risk mistakes on my part if, over the long term, I will learn and develop as a result of the experience	0.80	15 Tu jefe/a asume el riesgo de que te equivoques, si a largo plazo puedes aprender y desarrollarte gracias a tu iniciativa y experiencia
LEBQ16	16. I am encouraged to try out new ideas even if there is a chance they may not succeed	0.87	16 Te animan a que pruebes nuevas ideas, incluso si existe la posibilidad de que no tengan éxito
LEBQ17	17. My manager focuses on corrective action rather than placing blame when I make a mistake	0.79	17 Tu jefe/a se preocupa más de corregir lo que hayas hecho mal que en castigarte o reprenderte cuando cometes un error

*FL, standardized factor coefficient in sample 1 (Konczak et al., 2000).*

One sub-sample of 226 participants also completed the Minnesota Satisfaction Questionnaire (MSQ) short-form (Weiss et al., 1977; Fields, 2002; Stander and Rothmann, 2009) accessible at: http://vpr.psych.umn.edu/instruments/msq-minnesota-satisfaction-questionnaire. Given that there is no official version of the MSQ validated in Spanish, a back-translation process of the original English version was carried out by independent native translators (from English to Spanish and later from Spanish to English), and the correspondence between the original English version and the three non-official Spanish versions published in the official web page of the MSQ was shown. Likewise, Hirschfeld’s (2000) revised classification of intrinsic and extrinsic satisfaction for the MSQ was also used, as well as a general satisfaction item (Hackman and Oldham, 1980; Konczak et al., 2000).

In addition, another subsample of 590 people were asked if they had participated in the following programs to promote CI in the past 12 months (Juarez Tarraga et al., 2016): suggestion boxes, permanent team suggestion systems, short-term team suggestion systems, and self-directed work teams. Thus, for each of these people, we calculated the number of programs they had participated in (ranging from 0 to 4). This scale was previously used by Marin-Garcia et al. (2018), based on the works of Drehmer et al. (2000), Lawler et al. (2001), Juarez Tarraga et al. (2016), and Guerrero and Barraud-Didier (2004). Because the questions we ask are fairly objective and refer to four different exposures to easily identifiable CI programs, we think recall bias is not likely. Although the responses can be affected by respondents’ memory failure, this would affect the statistical power and reduce its effect on the criterion validity between CI and the LEBQ, which could be higher than the results indicate (Raphael, 1987).

### Data Analysis

The internal consistency of the items was analyzed through the inter-item correlation, corrected item-total correlation, and squared multiple correlation (Doval Dieguez and Viladrich Segués, 2011). Moreover, given that univariate kurtosis statistics were found to indicate non-normality, a CFA was performed using the Satorra–Bentler bias-corrected maximum-likelihood estimation method for the six-factor model proposed by Konczak et al. (2000) and for the one-dimensional model (null solution), in order to rule out the possibility that the LEBQ-Sp was measuring a unitary construct (Konczak et al., 2000; Llosa et al., 2017).

The cutoff values to consider an excellent fit were RMSEA ≤ 0.08, NFI, NNFI, IFI, and CFI ≥ 0.95 (Byrne, 2006; Finch and French, 2015). Cronbach’s alpha, compound reliability, and extracted variance (EV) were also calculated to assess convergent validity (cutoff values of 0.7 for the former and 0.5 for the latter) (Hair et al., 2009, 2018). Discriminant validity was assessed using the Fornell and Larcker (1981) criteria, and checking that 1 was not included in the correlation’s confidence intervals at 95% (Anderson and Gerbing, 1988; Bagozzi, 1994; Marin-Garcia and Conci, 2013).

To verify the relationship between EL and satisfaction, Pearson’s correlation coefficient was calculated. For the association between EL and CI, we performed an analysis of variance (ANOVA) with *post hoc* Bonferroni tests (Hair et al., 2009).

The SPSS 20 was used to compute descriptive statistics, correlation analyses, ANOVA, and internal consistency. The EQS version 6.3 program (Bentler, 2002) was used for CFA.

## Results

The descriptive analyses of the items (Table 2) show that none of the items on the Spanish version of the LEBQ have floor or ceiling effects. The mean values are located at the medium-low part of the scale (2.02–3.58), with a standard deviation between 1.32 and 1.69. On various items, the skewness and kurtosis are above or near the absolute value of 1, which could be considered a departure from normal distribution and suggest the use of Satorra–Bentler bias-corrected estimators.

**TABLE 2:** Descriptive statistics, standardized factor loadings of 17 LEBQ-sp items, and scales reliability.

	***N***	**Loadings**	**Minimum**	**Maximum**	**Mean**	**Standard deviation**	**Skewness**	**Kurtosis**	**Corrected item-total correlation**	**Squared multiple correlation**	**Cronbach’s alpha if item deleted**
F1	724	EV = 0.70 CR = 0.87 α = 0.87 mean inter-item correlations 0.689 (min 0.629; max 0.800)									
LEBQ01	736	0.891 f	0	5	3.26	1.382	–0.704	–0.230	0.801	0.670	0.771
LEBQ02	736	0.893^∗∗∗^	0	5	3.11	1.431	–0.620	–0.456	0.791	0.663	0.779
LEBQ03	729	0.708^∗∗∗^	0	5	3.31	1.327	–0.869	0.213	0.668	0.447	0.889
F2	711	EV = 0.54 CR = 0.78 α = 0.77 mean inter-item correlations 0.521 (min 0.401; max 0.666)									
LEBQ04	731	0.559 f	0	5	3.58	1.339	–0.922	0.212	0.491	0.254	0.800
LEBQ05	732	0.819^∗∗∗^	0	5	2.97	1.557	–0.480	–0.780	0.700	0.505	0.568
LEBQ06	723	0.801^∗∗∗^	0	5	2.83	1.556	–0.350	–0.865	0.628	0.450	0.657
F3	721	EV = 0.64 CR = 0.84 α = 0.84 mean inter-item correlations 0.632 (min 0.555; max 0.683)									
LEBQ07	732	0.757 f	0	5	3.03	1.497	–0.569	–0.614	0.663	0.454	0.811
LEBQ08	733	0.782^∗∗∗^	0	5	3.54	1.326	–0.995	0.489	0.678	0.486	0.793
LEBQ09	728	0.856^∗∗∗^	0	5	3.37	1.414	–0.886	0.088	0.760	0.580	0.711
F4	718	EV = 0.73 CR = 0.85 α = 0.84 mean inter-item correlations 0.724 (min 0.724; max 0.724)									
LEBQ10	731	0.867 f	0	5	3.35	1.369	–0.745	–0.088	0.724	0.524	n.a.
LEBQ11	725	0.845^∗∗∗^	0	5	3.36	1.359	–0.746	–0.076	0.724	0.524	n.a.
F5	711	EV = 0.59 CR = 0.81 α = 0.81 mean inter-item correlations 0.586 (min 0.511; max 0.712)									
LEBQ12	723	0.607 f	0	5	2.02	1.689	0.215	–1.245	0.565	0.320	0.832
LEBQ13	732	0.864^∗∗∗^	0	5	2.69	1.524	–0.277	–0.848	0.694	0.531	0.694
LEBQ14	732	0.806^∗∗∗^	0	5	2.70	1.559	–0.286	–0.941	0.711	0.546	0.674
F6	722	EV = 0.55 CR = 0.78 α = 0.78 mean inter-item correlations 0.545 (min 0.442; max 0.651)									
LEBQ15	734	0.779 f	0	5	2.84	1.494	–0.405	–0.750	0.705	0.504	0.611
LEBQ16	737	0.826^∗∗∗^	0	5	2.51	1.597	–0.152	–1.082	0.625	0.436	0.703
LEBQ17	726	0.593^∗∗∗^	0	5	3.10	1.439	–0.639	–0.396	0.540	0.307	0.788

*N listwise, 651; EV, extracted variance; CR, compound reliability; α, Cronbach’s alpha; n.a., not available calculation; f, fixed factor. ^∗∗∗^Estimated factor *p* < 0.001.*

The Cronbach’s alpha and compound reliability are >0.78 for each of the factors of the scale, and they were stable or decreased if an item was deleted (Table 2). The EV average was >0.50 in all cases.

Moreover, the correlations between the six scales are all significant with a medium or large size (Table 3). Following the Fornell and Larcker (1981) criteria, there are discriminant validity problems between F4 and F2; and between F6 and F3 and F5. Using the confidence interval test of the correlation, the discriminant validity problems disappear in the first case. Given that from 15 correlations only 3 show discriminant validity problems with one of the methods, we consider that the Spanish version shows enough discriminant validity.

**TABLE 3:** Descriptive scales and correlations and discriminant validity tests.

**Factor**	***N***	**Min.**	**Max.**	**Mean**	***SD***	**P25**	**P50**	**P75**	**F1**	**F2**	**F3**	**F4**	**F5**	**F6**
F1 delegation of authority	724	0.00	5.00	3.22	1.23	2.67	3.33	4.00	**0.835**	(0.35; 0.56)	(0.62; 0.96)	(0.42; 0.73)	(0.50; 0.80)	(0.52; 0.86)
F2 accountability	711	0.00	5.00	3.12	1.22	2.33	3.33	4.00	0.460	**0.736**	(0.40; 0.62)	(0.60; 0.89)	(0.56; 0.79)	(0.43; 0.64)
F3 self-directed decision making	721	0.00	5.00	3.30	1.22	2.67	3.67	4.00	0.798	0.515	**0.799**	(0.54; 0.88)	(0.61; 0.92)	(0.67; 1.02)
F4 information sharing	718	0.00	5.00	3.35	1.27	2.50	3.50	4.00	0.578	0.745	0.713	**0.856**	(0.59; 0.91)	(0.50; 0.81)
F5 skill development	711	0.00	5.00	2.47	1.35	1.33	2.67	3.33	0.649	0.678	0.762	0.746	**0.767**	(0.69; 1.01)
F6 coaching for innovative performance	722	0.00	5.00	2.81	1.26	2.00	3.00	3.67	0.688	0.533	0.845	0.654	0.853	**0.740**

	***N***	**Cronbach’s α**			**Rho**				**F1**	**F2**	**F3**	**F4**	**F5**	**F6**

General satisfaction	226	–			–				0.455	0.402	0.430	0.468	0.478	0.349
Intrinsic satisfaction	226	0.866			0.868				0.621	0.231	0.564	0.456	0.510	0.520
Extrinsic satisfaction	226	0.815			0.818				0.464	0.659	0.498	0.683	0.655	0.598

*Descriptive results (SPSS): P25, percentile 25; P50, median; P75, percentile 75; SD, standard deviation. Correlations (based on EQS outputs): diagonal (bold), VE squared root; diagonal inferior, Pearson correlation (all correlations significant at the 0.001 level; two-tailed); diagonal superior, correlations confidence interval 95%; *N* = 651. Last three rows, Pearson correlation (all correlations significant at the 0.001 level; two-tailed).*

The one-factor and six-factor models were tested by CFA, using the maximum-likelihood estimation method. The results for the model with one-factor are: Satorra–Bentler Chi-square divided by degrees of freedom (SB-χ^2^/*df*) = 10.05; NFI = 0.769; NNFI = 0.756; IFI = 0.787; CFI = 0.787; and RMSEA = 0.118; and for the model with six-factors are: SB-χ^2^/*df* = 3.31; NFI = 0.934; NNFI = 0.938; IFI = 0.953; CFI = 0.952; and RMSEA = 0.060. With the criteria adopted, the one-factor model did not show an acceptable fit (all of the indices were out of bounds), whereas the six-factor model showed a good fit.

Criterion validity of the six-factor model was assessed by comparing correlations with job satisfaction (Hirschfeld, 2000) in a sub-sample of 226 participants who also completed the MSQ short form. All the factors showed significant positive correlations (*p* ≤ 0.001) with all the facets of job satisfaction: intrinsic, extrinsic, and general satisfaction. Most of the correlations are of medium intensity, with values between 0.40 and 0.69, with two exceptions: between F2 and intrinsic satisfaction (0.231), and between F6 and general satisfaction (0.349). These results support the adequacy of the scale in terms of its relationship with other theoretically relevant variables (Konczak et al., 2000; Stander and Rothmann, 2009).

Furthermore, based on the ANOVA, we can conclude that there is a clear association between the number of CI programs in which an employee participates and the supervisor’s EL style (Table 4). When employees do not participate in formal CI programs, or participation is based on suggestion-box type programs with individual contributions (which are common when a person participates in less than two formal programs), the leader tends to display fewer behaviors of delegation of authority, accountability, self-directed decision-making, information sharing, skill development, and coaching for innovative performance, than when employees participate in three or four formal CI programs.

**TABLE 4:** Relationships between empowering leadership and continuous improvement.

**Factor**	**CIp**	***N***	**Mean**	**Bonferroni test**	***SD***	**Std. Error**	**Lo95**	**Hi95**	**Min.**	**Max.**	**ANOVA**
LEBQ1	0	229	2.99	2+; 3^∗∗^	1.28	0.08	2.83	3.16	0.00	5.00	8.49^∗∗^
	1	213	3.17	3^∗∗^	1.21	0.08	3.00	3.33	0.00	5.00	
	2	92	3.40	0+; 3+	1.07	0.11	3.18	3.62	0.00	5.00	
	3	45	4.00	0^∗∗^; 1^∗∗^; 2+	0.89	0.13	3.73	4.27	1.67	5.00	
	4	11	3.91		0.65	0.20	3.47	4.35	3.00	5.00	
LEBQ2	0	229	2.91	1+; 3^∗∗^; 4^∗∗^	1.21	0.08	2.75	3.07	0.00	5.00	7.23^∗∗^
	1	213	3.21	0+; 4^∗^	1.23	0.08	3.04	3.37	0.00	5.00	
	2	92	3.25	4^∗^	1.14	0.12	3.01	3.49	0.00	5.00	
	3	45	3.58	0^∗∗^	1.05	0.16	3.26	3.89	0.67	5.00	
	4	11	4.42	0^∗∗^; 1^∗∗^; 2^∗∗^	0.45	0.14	4.12	4.73	3.67	5.00	
LEBQ3	0	229	3.00	1^∗^; 2^∗∗^; 3^∗∗^; 4^∗^	1.33	0.09	2.83	3.18	0.00	5.00	9.57^∗∗^
	1	213	3.38	0^∗∗^; 3^∗∗^	1.16	0.08	3.22	3.53	0.00	5.00	
	2	92	3.46	0^∗^; 3+	1.01	0.11	3.25	3.67	0.67	5.00	
	3	45	4.02	0^∗∗^; 1^∗∗^; 2+	0.85	0.13	3.77	4.28	1.33	5.00	
	4	11	4.03	0^∗∗^	0.55	0.16	3.66	4.40	3.00	5.00	
LEBQ4	0	229	3.13	3^∗∗^	1.33	0.09	2.96	3.30	0.00	5.00	5.86^∗∗^
	1	213	3.39	3^∗∗^	1.23	0.08	3.22	3.55	0.00	5.00	
	2	92	3.50		1.13	0.12	3.27	3.73	0.00	5.00	
	3	45	3.96	0^∗∗^; 1^∗∗^	1.07	0.16	3.63	4.28	1.00	5.00	
	4	11	4.09		0.83	0.25	3.53	4.65	2.50	5.00	
LEBQ5	0	229	2.16	2^∗∗^; 3^∗∗^; 4^∗∗^	1.34	0.09	1.98	2.33	0.00	5.00	11.68^∗∗^
	1	213	2.44	3^∗∗^; 4^∗∗^; 5^∗∗^	1.29	0.09	2.27	2.62	0.00	5.00	
	2	92	2.74	0^∗∗^	1.19	0.12	2.49	2.98	0.00	5.00	
	3	45	3.30	0^∗∗^; 1^∗∗^	1.18	0.18	2.95	3.66	1.00	5.00	
	4	11	3.70	0^∗∗^; 1^∗^	1.04	0.31	3.00	4.39	1.33	4.67	
LEBQ6	0	229	2.58	2^∗∗^; 3^∗∗^; 4^∗∗^	1.30	0.09	2.41	2.75	0.00	5.00	9.40^∗∗^
	1	213	2.83	3^∗∗^	1.14	0.08	2.68	2.99	0.00	5.00	
	2	92	3.14	0^∗∗^	1.06	0.11	2.92	3.36	0.00	5.00	
	3	45	3.52	0^∗∗^; 1^∗∗^	1.17	0.17	3.17	3.87	0.33	5.00	
	4	11	3.73	0^∗∗^	0.55	0.17	3.36	4.10	3.00	4.67	

*CIp, number of continuous improvement programs in which employee was involved in the last 12 months; Bonferroni test, significant mean differences (Bonferroni corrected) between the number of CIp of the row and the number of CI programs indicated in the cell: ^∗∗^*p* < 0.01, ^∗^*p* < 0.05, +*p* < 0.10; SD, standard deviation; Lo95, 95% confidence interval for mean lower bound; Hi95, 95% confidence interval for mean upper bound.*

Even so, differences appear between the factors that make up the LEBQ and the degree of participation in programs to promote CI (there is a statistically significant difference between the values from LEBQ1 to LEBQ4 and from LEBQ5 and LEBQ6 of almost one point). It seems that when a leader has to work with collaborators who are more active in CI (through more formal initiatives, not only individual, but also group), s/he shows more frequent and more homogeneous EL behaviors in the six factors of the LEBQ. However, in contexts that are “poorer” in CI programs (zero or one program), the leaders focus on the dimensions of delegation of authority, accountability, self-directed decision-making, and information sharing, whereas they show fewer behaviors of coaching for innovative performance and, above all, skill development toward their subordinates.

From another perspective, information sharing is the dimension that seems to be least affected by the number of CI programs in which an employee participates. The mean values in this dimension are usually among the highest, and the differences on this variable are the smallest. It seems to be a behavior that is easier for the leader to perform than the other behaviors that measure LEBQ.

Likewise, it is interesting to point out that almost three-fourths of the employees in our sample work in “poor” CI settings, so that there is considerable room for improvement. Thus, it becomes a challenge to develop all the behaviors linked to EL in leaders, especially those related to coaching for innovative performance and skill development.

## Discussion

Most of the instruments designed to measure empowerment are in English, and most of the research is carried out in developed countries (Seibert et al., 2011; Sharma and Kirkman, 2015; Lee et al., 2018). In order to make progress in developing empowerment in other geographical contexts, such as Spanish speaking countries, including some developing countries, it is necessary to propose new instruments or adapt the ones already validated in English to Spanish, which is the option chosen in this study.

The present article describes the Spanish adaptation of one of the best existing scales to promote employee empowerment: the LEBQ, developed by Konczak et al. (2000). For this purpose, a broad sample of employees from companies with CI processes in place is used. Moreover, the study examines the relationships between EL and job satisfaction and different programs related to CI.

The results show that the LEBQ-sp has good psychometric properties because it reproduces the proposed six-factor theoretical model, and the subscales show satisfactory reliabilities and sufficient convergent and discriminant validity. In addition, all the subscales are positively related to job satisfaction, contributing new evidence about its validity.

The one-dimensional model does not receive support, as occurred in the study by Konczak et al. (2000), and unlike what was proposed by other authors such as Stander and Rothmann (2009). The scales of the LEBQ, in its Spanish version, are positively related to job satisfaction, although not all the studies have used the MSQ. The original study by Konczak et al. (2000), for example, used a single item to measure general job satisfaction. Furthermore, the sample in our study was obtained from a varied set of companies (with different degrees of implementation of CI processes), making it possible to overcome one of the limitations of the study by Konczak et al. (2000), which was carried out in a single organization. Stander and Rothmann (2009) also used the MSQ in their research (although with a very limited sample size, as the authors recognize), reaching the conclusion that the two constructs are related. However, these authors treated the LEBQ as if it were one-dimensional, even though, based on the results of the present study, we can confirm that a six-dimensional model fits significantly better.

After establishing the validity of the LEBQ as a diagnostic instrument, we were able to identify a clear association between EL and the number of formal CI programs in which employees participate. As other authors have highlighted, in order to promote greater implementation of CI in organizations, it is necessary to achieve a greater degree of empowerment among the employees (Fryer et al., 2007; Poksinska et al., 2013; Hirzel et al., 2017; Jurburg et al., 2017). In this regard, it seems clear that, as companies invest more in formal CI programs, the leaders have to adapt their behavior to show more delegation of authority, transmit accountability, transfer decision-making to employees, and share information with them, and, especially, promote subordinates’ skill development and coach them in innovative performance (Costa Nogueira et al., 2018).

Given that many supervisors have medium or low levels of EL in general, and they are especially low in developing their collaborators’ talent or guiding them in the innovation process, new questions arise for future research, such as: What guidelines can we propose for organizations that need to implement CI programs? This evolution depends not only on unfolding technical aspects, but it must also be accompanied by the development of personal and social competencies in all the supervisors and employees (Garcia-Sabater et al., 2012).

Our study also has some limitations. The data were gathered only from questionnaires, which could lead to problems with common method variance. In addition, the purposive sample does not allow us to offer normative data on the Spanish or Latin America population, and this limitation should be resolved in future research. A further limitation results from the measurement method used to determine CI because it depends on the respondent’s ability to provide information from the last 12 months without recall bias or memory failure. However, from an applied management perspective (Konczak et al., 2000; Zigun, 2016), the LEBQ-sp can be a practical tool for providing feedback and coaching managers on their use of leader behaviors associated with empowerment in organizations.

## Conclusion

In conclusion, this paper validated the Spanish version of the LEBQ scale, which is an established instrument used to measure leaders’ empowering behaviors. The CFA supported the original structure of the LEBQ scale. The data gathered to examine the reliability of the LEBQ-sp reveal that the LEBQ-sp shows a high level of internal consistency, as well as sufficient convergent and discriminant validity. Moreover, the results indicate that more EL is related to greater employee work satisfaction, and more CI is related to more EL.

Finally, scholarly and practical evidence indicates that organizations that use empowering initiatives outperform their counterparts that rely more on traditional hierarchical structures (Sharma and Kirkman, 2015). Taking the demands of an increasingly dynamic and complex environment (innovation, globalization, etc.) into account, it is currently considered imperative for leaders to promote empowerment initiatives among their employees. Our study provides Spanish-speaking researchers and professionals with an instrument to measure the degree to which leaders accept this responsibility.

## Data Availability Statement

The datasets generated for this study are available on request to the corresponding author.

## Ethics Statement

This study was carried out in accordance with the ethical guidelines of the American Psychological Association and the Declaration of Helsinki, and it was reviewed and approved by the Ethics Committee of the Polytechnic University of Valencia. The patients/participants provided their written informed consent to participate in this study.

## Author Contributions

TB and JM-G contributed conception and design of the study, and gave the final approval of the manuscript before the submission. JM-G organized the database and performed the statistical analysis. TB wrote the first draft of the manuscript. Both authors reviewed it critically.

## Conflict of Interest

The authors declare that the research was conducted in the absence of any commercial or financial relationships that could be construed as a potential conflict of interest.
